# Supplementary material for: Small-scale livelihood and cultural fire: Global spatiotemporal characteristics, and gaps in data
Source: PLoS One. 2026 Jan 20;21(1):e0339561. doi: 10.1371/journal.pone.0339561 (PMC12818736; doi:10.1371/journal.pone.0339561)
Supplement: S1 Appendix — (DOCX) [file pone.0339561.s001.docx]

| ***Fire use purpose*** | | ***Reason*** | ***Description*** | ***N*** |
| --- | --- | --- | --- | --- |
| *Higher-tier* | *Lower-tier* |  |  |  |
| **Agriculture (A)**  Fire is used in agriculture for the initial clearance of vegetation to establish agricultural plots, as well as during cycles of planting and harvest to maintain a plot. Examples in LIFE of burning to clear vegetation to establish agricultural plots are distributed across the equatorial tropics. Examples of post-harvest crop residue / weed burning in LIFE are concentrated in southern and south-east Asia, though it is also particularly widespread in northern and central Asia and eastern Europe. | A1. Clear vegetation for swidden or semi-permanent agriculture | Primary purpose | Fire clears vegetation between cycles of alternating cropping and fallow, supplying nutrients to the soil. Chopped and dried vegetation is burned in piles or fire is broadcast across the plot. In areas with low soil fertility, additional vegetation may be taken from surrounding areas, to increase organic matter supplied to the soil. | 325 |
|  | A2. Clear vegetation for permanent agriculture | Primary purpose | Fire clears vegetation to establish permanent plots of perennial or annual crops, possibly after one or more cycles of swidden. Similar process to swidden clearance. | 46 |
|  | A3. Clear weeds and/or crop residues during the growing season | Primary purpose | Includes various practices, e.g., burning weeds in piles during the growing season or burning sugarcane plants before harvest to facilitate faster harvesting. | 22 |
|  | A4. Clear weeds and/or crop residues after harvest to enable planting | Primary purpose | In permanent or semi-permanent agriculture where annual crops are grown repeatedly on the same plot, burning after harvest clears crop residues and/or weeds to rapidly prepare for the next crop. Fire may be broadcast across the whole plot, or material is burned in piles. | 133 |
|  | A5. Reduce crop pests | Primary purpose or co-benefit | Fires set for other agricultural purposes can also reduce crop pests but is sometimes used specifically for this purpose during the growing season, e.g., through removing vegetative cover used by pests. | 34 |
| **Pastoralism (P)**  These fire uses are associated with establishing and maintaining pasture or encouraging movement of livestock. Includes practices associated with extensive pastoralism in which livestock are grazed on natural vegetation and tend to range over large areas and more intensive pastoralism in which livestock are grazed in smaller areas of improved grassland. Examples in LIFE span the continents. | P1. Clear vegetation to establish new pasture areas | Primary purpose | Like clearance for swidden or permanent cropping, with vegetation cut and dried before burning. May take place in areas that have previously been cropped. | 18 |
|  | P2. Enhance forage for grazing livestock | Primary purpose | Burning maintains forage, in the longer term by suppressing woody growth, in the shorter term by encouraging fresh growth of grasses, forbs, and/or leafy vegetation. In extensive pastoral systems, fires extinguish where they meet previously burned areas, or natural firebreaks. In more intensive systems, artificial firebreaks may be constructed to limit fire extent. | 219 |
|  | P3. Herd livestock | Primary purpose or co-benefit | Sometimes a co-benefit of burning to enhance forage, as this encourages movement of livestock. Sometimes fire/smoke are directly used to drive livestock. | 17 |
|  | P4. Reduce livestock pests and predators | Co-benefit | A common co-benefit of fires set to enhance forage, pests are also burned (or their vegetative cover reduced). | 50 |
| **Hunting and fishing (HF)**  Fire is used for various reasons to prepare for, or during, hunting or fishing. Examples of these fires in LIFE are distributed throughout the Americas, Africa, southern and southeast Asia, and Australasia. | HF1. Create or improve habitat for hunted or fished species | Primary purpose or co-benefit | On inter-annual timescales, fire maintains habitat for hunted or fished species. | 24 |
|  | HF2. Renew forage to draw hunted or fished species into a particular area | Primary purpose or co-benefit | Fire renews forage for animals, drawing them into certain areas where they can be located for hunting/ fishing on weekly to monthly timescales. | 67 |
|  | HF3. Improve visibility or access specifically for hunting or fishing | Primary purpose or co-benefit | Burning clears vegetative cover rendering hunted/ fished species, or their tracks, more visible, or improves access to hunting/ fishing grounds. | 69 |
|  | HF4. Drive animals when hunting | Primary purpose | Fire or smoke force animals in certain directions for hunting. Commonly practiced during communal hunts. | 65 |
|  | HF5. Kill, injure, or tire animals when hunting | Primary purpose | Fire or smoke are used as a weapon during hunting. | 12 |
| **Gathering (G)**  Fire is used for various reasons to aid the gathering of foraged resources or improve their quality or quantity. Examples in LIFE are distributed across the Americas, Africa, southern and southeast Asia, Australasia and Europe. | G1. Enhance productivity of foraged resources | Primary purpose or co-benefit | Burning enhances the quality and productivity of foraged resources in various ways. Sometimes, fire stimulates certain phenological stages, e.g., germination, flowering, fruiting. Where plants resprout after fire this can bring more vigorous growth, or desired growth forms. Burning can also reduce competing vegetation or pests. | 134 |
|  | G2. Ease the collection of a foraged resource by improving visibility or access | Primary purpose or co-benefit | Burning clears vegetation making it easier to access/ see a foraged resource. | 42 |
|  | G3. Drive wild bees away from hives for honey collection | Primary purpose | Smoke drives bees away from their hives. | 49 |
| **Charcoal and fuelwood production (C)**  Burning to produce charcoal or fuelwood. Relatively few examples in LIFE, with these being concentrated in Africa. | C1. Produce charcoal | Primary purpose | To produce charcoal, felled trees are buried in mounds or pits and smouldered for several days. | 17 |
|  | C2. Produce fuelwood for gathering, or enable gathering of fuelwood | Primary purpose or co-benefit | Burning can produce fuelwood by provoking tree mortality or hastening drying of deadwood. | 20 |
| **Movement (M)**  Burning to improve general landscape access. Examples in LIFE are distributed across the continents. | M1. Maintain and open trails and waterways for general access | Primary purpose or co-benefit | Fire improves access by clearing trails or waterways or reducing dense vegetation across wider landscape patches (in this case improved access is likely a co-benefit of burning for other reasons). | 51 |
| **Human health & wellbeing (HW)**  Beyond subsistence, there are a variety of reasons for burning associated with promoting landscapes that support human health and wellbeing. These include fires that reduce wildfire risk, the risk of danger from animals, or that promote aesthetically pleasing landscapes. Examples in LIFE span the continents, but are most common in Africa, South America, and Australia. | HW1. Reduce animals that are dangerous to or unwanted by humans | Co-benefit | Burning reduces habitat/ vegetative cover for unwanted animals (e.g., snakes, ticks) or kills them directly, but this is not often a primary reason. | 64 |
|  | HW2. Reduce fuel loads to reduce risk of wildfires at a landscape scale | Primary purpose, or co-benefit | Progressive burning of landscape patches fragments the fuel landscape, reducing wildfire risk. | 88 |
|  | HW3. Create firebreak using fire to protect e.g., resources, farms, sacred sites | Primary purpose, or co-benefit | Targeted, delimited, burning protects specific entities (settlements, farms, sacred sites, resources) from wildfire. | 47 |
|  | HW4. Suppress a wildfire (using backing fire to fight fire with fire) | Primary purpose | Controlled backing fire (set against the wind or upslope) meets a wildfire front, and the fires extinguish one another. | 6 |
|  | HW5. Produce a more aesthetically pleasing landscape, or for enjoyment | Co-benefit | In many cultures the ‘cleaner’ post-fire landscape is considered more beautiful and burning may be seen as an enjoyable activity. | 24 |
| **Social signals (S)**  This category includes fires that are set because of their social significance, to communicate messages, in ritual or celebration, to protest or assert rights or cultural identity. Examples in LIFE span the continents. | S1. Communicate about current activity | Primary purpose | Fire/smoke signal to comrades about current activity, e.g., success of hunt, or broken-down vehicle. | 17 |
|  | S2. Show disapproval or protest (arson) | Primary purpose | Burning in resistance to or dispute with the state, landowners, or other community members, e.g., over land access or protected areas regulations. | 53 |
|  | S3. For ritual or ceremonies | Primary purpose | Burning in rituals or ceremonies occurring in the landscape outside settlements. | 16 |
|  | S4. Assert or maintain cultural identity | Co-benefit | Burning may persist or be revived as a way of expressing cultural identity. Not usually the sole reason for burning – fire usually retains role in subsistence/ wildfire mitigation. | 113 |
