# Supplementary material for: Small-scale livelihood and cultural fire: Global spatiotemporal characteristics, and gaps in data
Source: PLoS One. 2026 Jan 20;21(1):e0339561. doi: 10.1371/journal.pone.0339561 (PMC12818736; doi:10.1371/journal.pone.0339561)
Supplement: S2 Appendix — (DOCX) [file pone.0339561.s002.docx]

**Supplementary information file 2 to accompany the article ‘Small-scale livelihood fire: global spatiotemporal characteristics, and gaps in data’**

Supplementary figures S1 and S2 show case study locations of fire use practices with known purpose(s) for which spatiotemporal data was available, in the same format as Fig 1 of the main text, but enlarged over Africa (Supplementary Fig. S1) and south-east Asia (Supplementary Fig. S2), to better display these regions where the density of case studies is very high.

Supplementary figures S3, S4, S5 show mean monthly precipitation and/or potential evapotranspiration (PET) for all case study locations of fire use practices analysed for seasonality of burning.

PET and precipitation data were sourced from ERA5 reanalysis products (Hersbach H, Bell B, Berrisford P, Hirahara S, Horányi A, Muñoz‐Sabater J, et al. The ERA5 global reanalysis. Quarterly Journal of the Royal Meteorological Society. 2020; 146(730): 1999-2049. <https://doi.org/10.1002/qj.3803>).

Seasonality type was determined from the ERA5 PET and precipitation using the ‘seasonality cause’ metric of Feddema (2005) (Feddema JJ. A revised Thornthwaite-type global climate classification. Physical Geography. 2005; 26(6): 442-466. <https://doi.org/10.2747/0272-3646.26.6.442>).


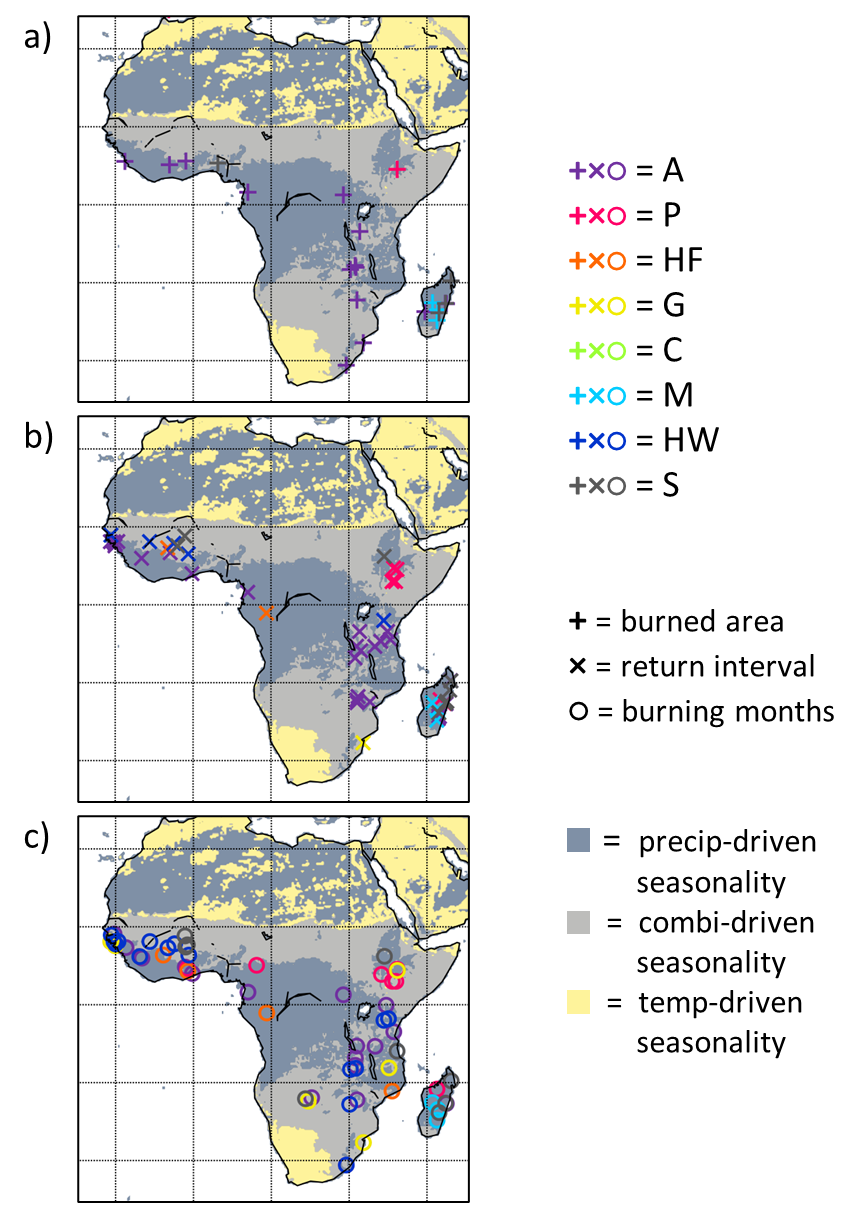


**Supplementary Fig. S1** | Enlargement of Fig 1 (main text), showing case study locations in Africa of fire use practices with known purpose(s) for which spatiotemporal data was available, separated by the type of data available at each case study location: a) locations with burned area data; b) locations with return interval data; c) locations with data on months when burning takes place. Background shading indicates seasonality type. The colour of points indicates higher-tier fire use purpose (A = agriculture, P = pastoralism, HF = hunting and fishing, G = gathering, C = charcoal and fuelwood production, M = movement, HW = health and wellbeing, S = social signals). Where a case study described multiple fire use purposes at the same location, fire use purposes were plotted in the order listed above and so only the last fire use purpose is visible.


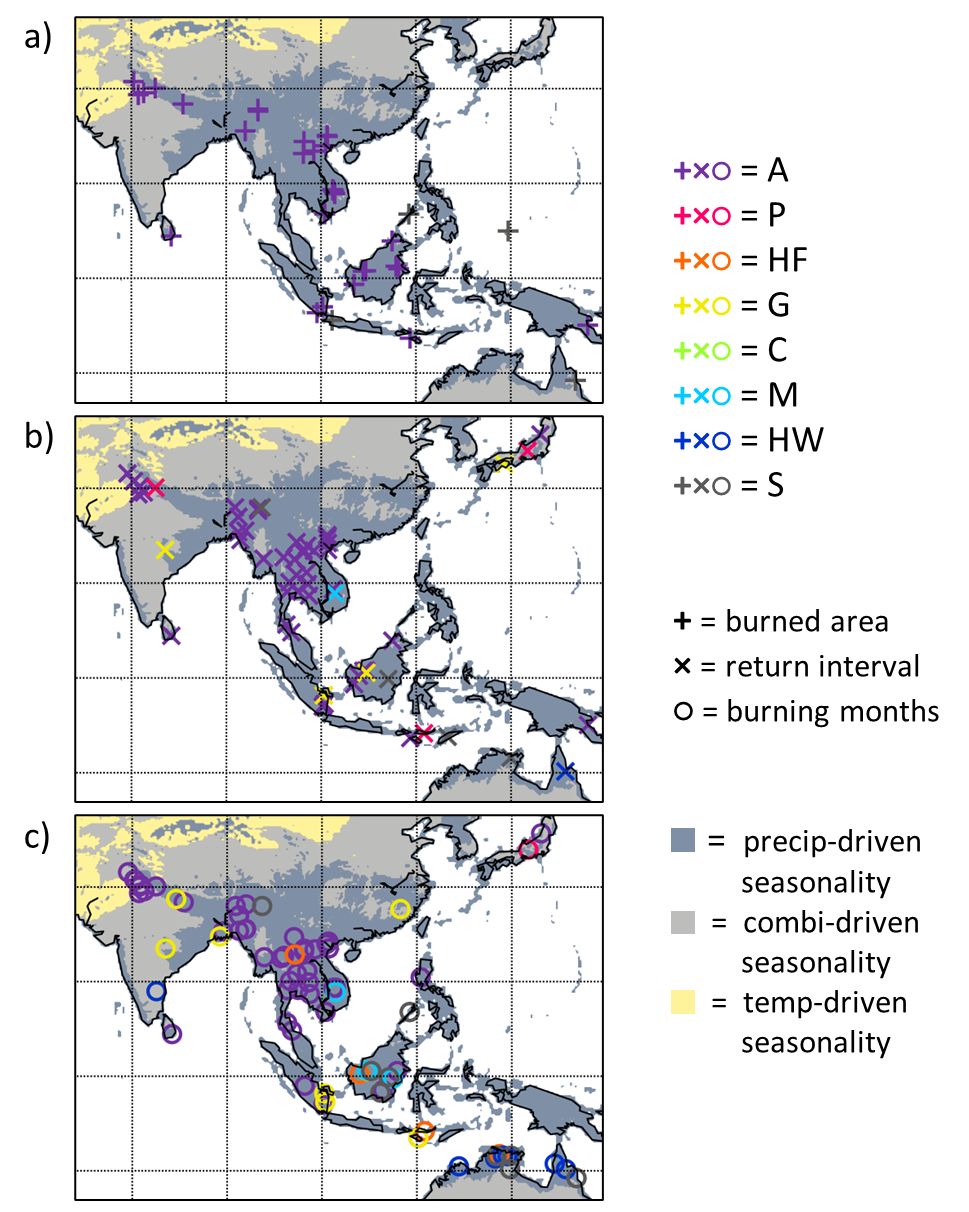


**Supplementary Fig. S2** | Enlargement of Fig 1 (main text), showing case study locations in south-east Asia of fire use practices with known purpose(s) for which spatiotemporal data was available, separated by the type of data available at each case study location: a) locations with burned area data; b) locations with return interval data; c) locations with data on months when burning takes place. Background shading indicates seasonality type. The colour of points indicates higher-tier fire use purpose (A = agriculture, P = pastoralism, HF = hunting and fishing, G = gathering, C = charcoal and fuelwood production, M = movement, HW = health and wellbeing, S = social signals). Where a case study described multiple fire use purposes at the same location, fire use purposes were plotted in the order listed above and so only the last fire use purpose is visible.


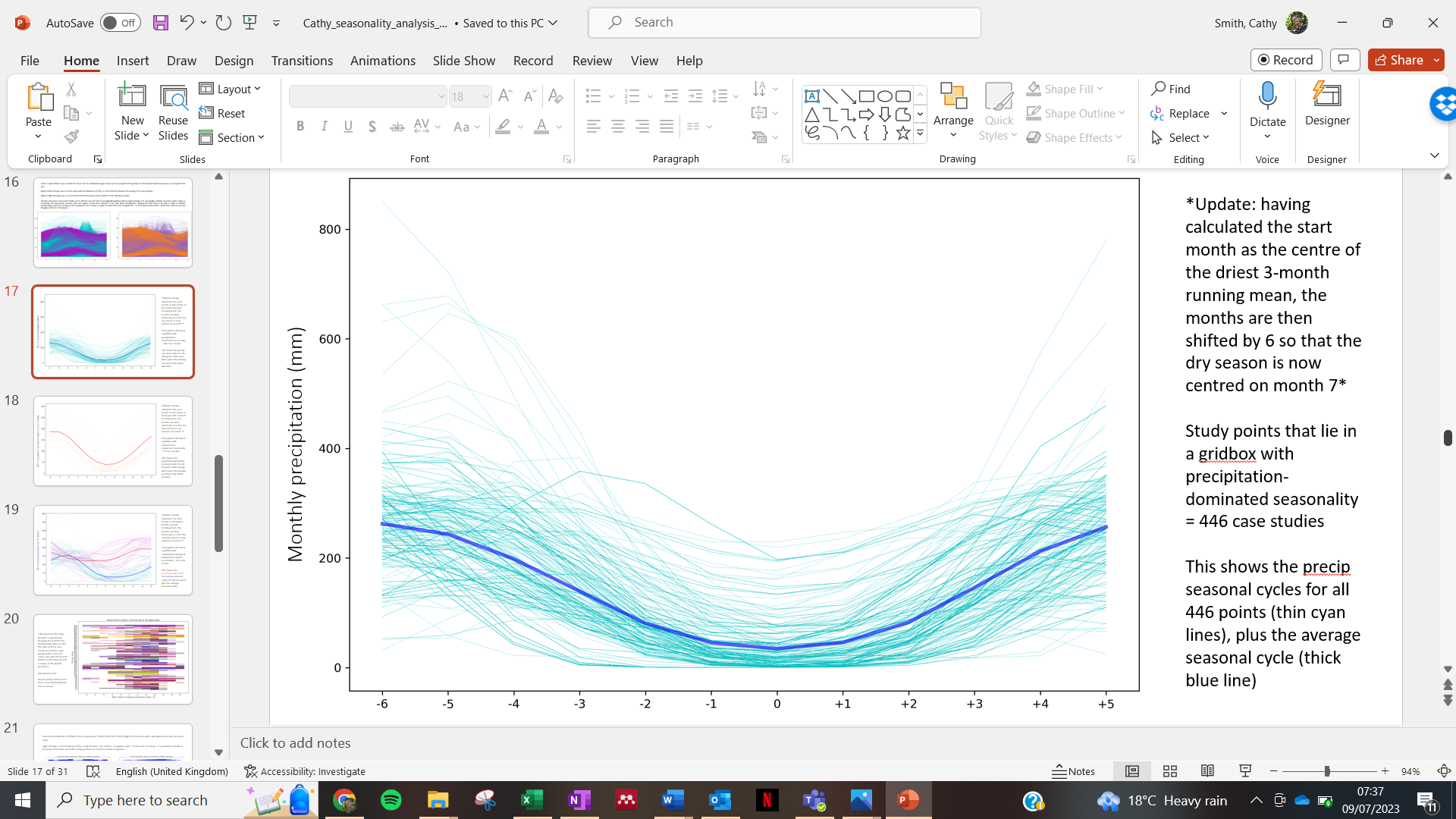


**Supplementary Fig. S3** | Mean monthly precipitation for the period 1990-2020 for all case study locations of fire use practices in locations with precipitation-driven climate seasonality and for which seasonality of burning was analysed. Dark line indicates mean monthly precipitation across all case study locations. Precipitation curves are aligned so that on the x-axis, 0 = centre of the driest 3-month period.


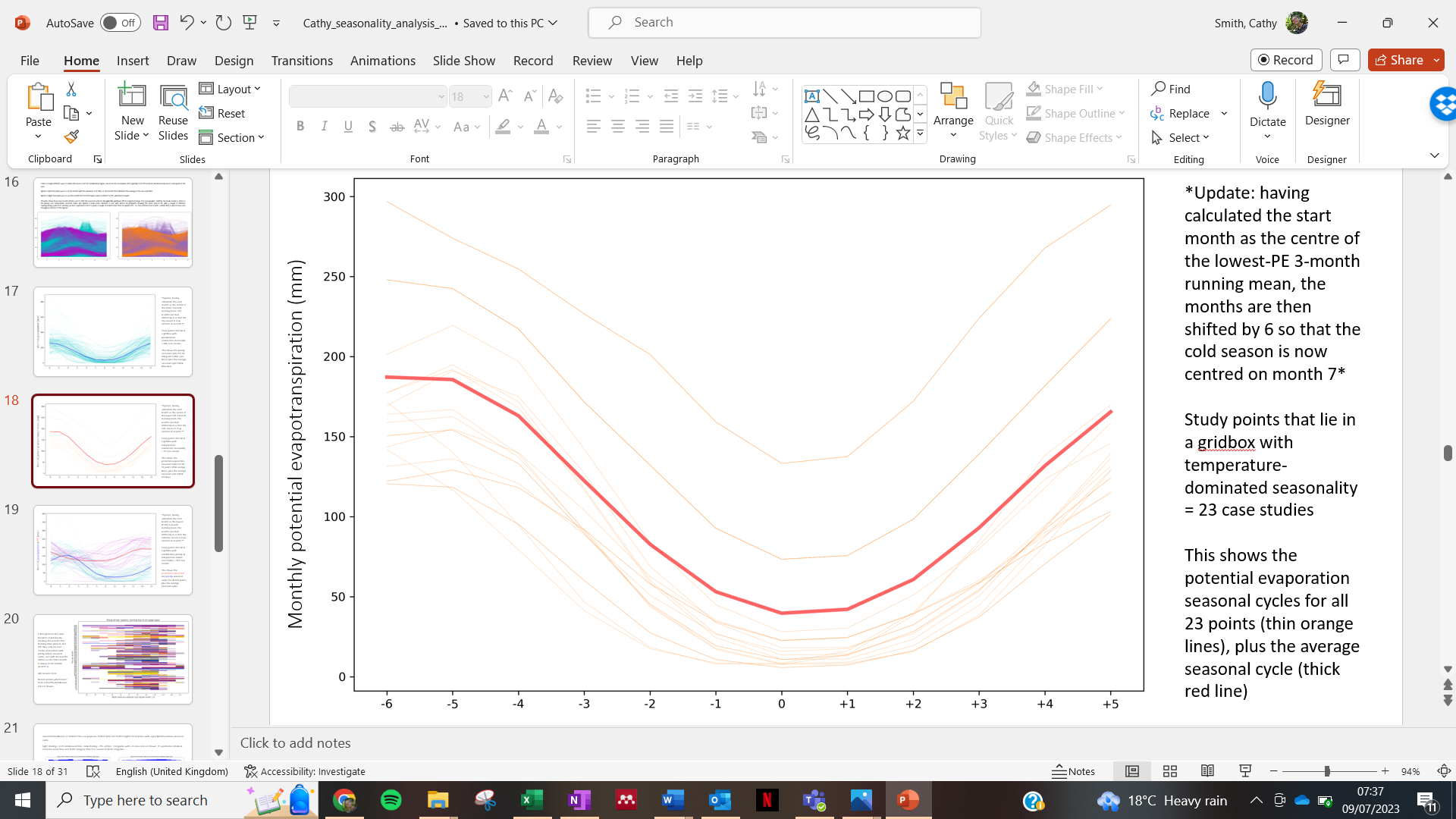


**Supplementary Fig. S4** | Mean monthly PET for the period 1990-2020 for all case study locations of fire use practices in locations with temperature-driven climate seasonality and for which seasonality of burning was analysed. Dark line indicates mean monthly PET across all case study locations. PET curves are aligned so that on the x-axis, 0 = centre of the 3-month period with the lowest potential evapotranspiration.


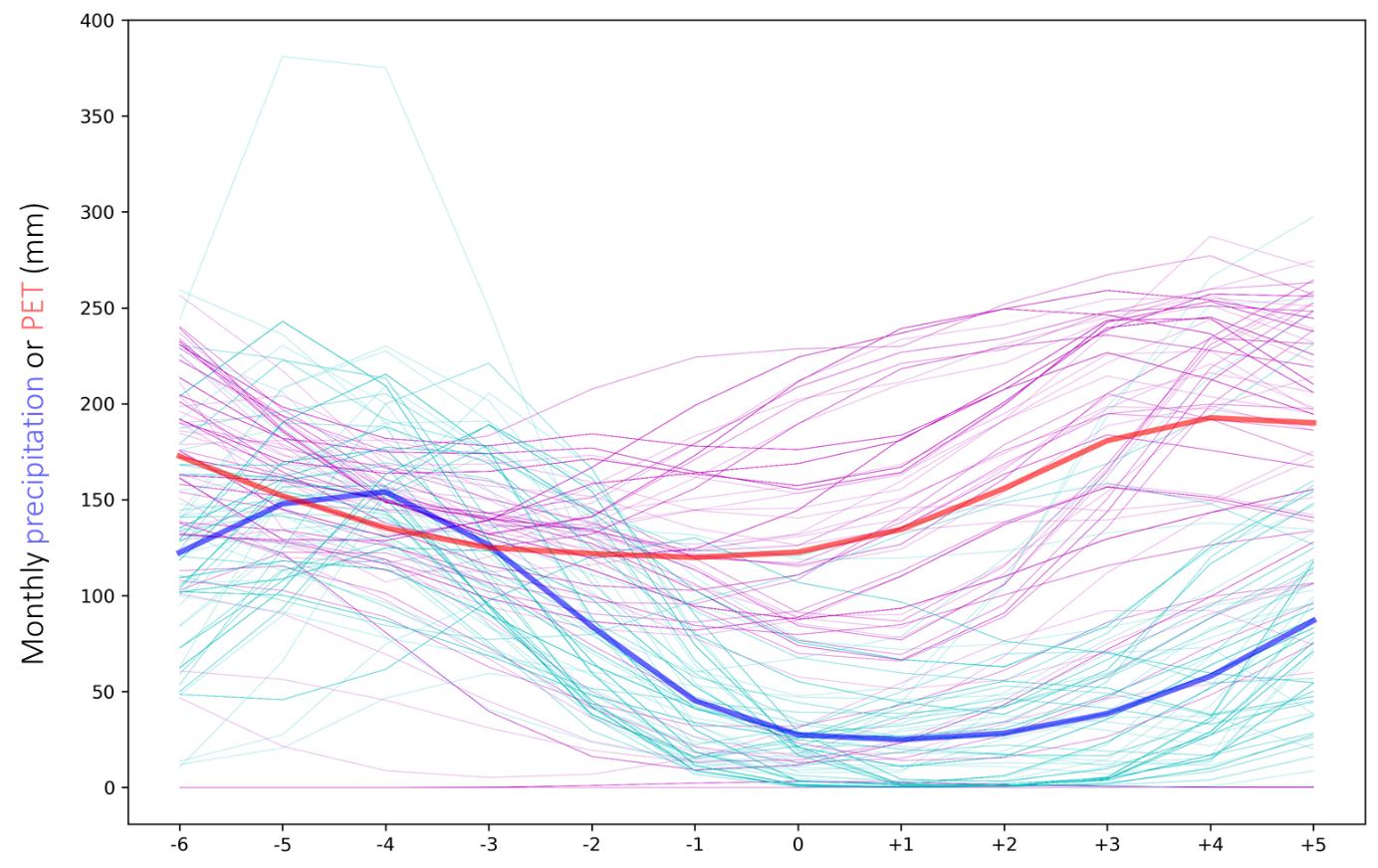


**Supplementary Fig. S5** | Mean monthly precipitation and PET for the period 1990-2020 for all case study locations of fire use practices in locations with combination-driven climate seasonality and for which seasonality of burning was analysed. Dark lines indicate mean monthly precipitation and PET across all case study locations. Precipitation and PET curves are aligned so that on the x-axis, 0 = centre of the 3-month period with the lowest sum of precipitation and PET.
