# Supplementary material for: Small-scale livelihood and cultural fire: Global spatiotemporal characteristics, and gaps in data
Source: PLoS One. 2026 Jan 20;21(1):e0339561. doi: 10.1371/journal.pone.0339561 (PMC12818736; doi:10.1371/journal.pone.0339561)
Supplement: Attachment — Submitted filename: PONE-D-24-10824_R1_author_response_to_comments.docx [file pone.0339561.s004.docx]

PONE-D-24-10824

Small-scale livelihood and cultural fire: global spatiotemporal characteristics, and gaps in data

**Author responses to Reviewer comments**

*We are very grateful to the two reviewers for their critical appraisal of our manuscript and suggestions for how it could be improved, particularly with regard to the structure and framing of the Results and Discussion, as well as highlighting some instances where the manuscript would benefit from more precise clarification of the data and methods.*

*In response to the two reviews, we have comprehensively re-written the Results section with an entirely new structure, as well as re-writing substantial portions of the Introduction, Methods, and Discussion to better frame the motivation and relevance of our analysis, as well as provide clearer and more consistent descriptions of the published dataset we employ.*

*Below, we have responded to each of the reviewers’ comments in detail; the reviewer comments are in normal text with our responses in cyan italic text. Where we give line numbers of the changes we have made in our responses, these refer to the clean version of the revised manuscript; a version with (extensive!) tracked changes is also provided.*

**Reviewer #1:**

I am sure there is a great paper to come from the LIFE database and wish you luck with that. I do not think you have achieved it here for the reasons explained below. At the end I suggest a way you could rework the contribution and arrive on a more robust contribution.

*We appreciate the comments. We have taken many of them on board and have substantially revised the manuscript as a result. However, we also believe some of these comments arise from misunderstandings of the content and dataset being analysed. We have tried to clarify these below, and where possible, to revise the text for additional clarity. The comments on the Results section were particularly helpful and we have fundamentally restructured this. Similarly, comments on the Introduction helped to sharpen the text, and comments on the Discussion highlighted where we could be more precise about the contribution of our data to wider scientific efforts. However, comments on the Methods suggest some misunderstandings and we respond to these below and in changes to the manuscript text to improve clarity. Overall, we thank the reviewer for their engagement with our work.*

ABSTRACT

What are small-scale livelihoods? A definition of this conception would be useful. Later it says those that do not rely on labour, yet this leaves important characteristics omitted (e.g. size of holding, market or subsistence orientation, traditional, Indigenous, colonist etc). Further many small scale farmers do exchange labour, and or pay for daily labour at certain times of the year, or if migration has taken place in the household etc.. would these then be omitted?

Unclear how or why collecting more data will be useful.

*We agree that a clear definition of these terms is useful. However, we feel the abstract is not the best place to unpack this given word limits and much other content to address. Instead, we define the concept in the opening paragraph of the Introduction. To enhance clarity, we have now split this into two sentences [L55-62] as:*

“…controlled fire use retains an important cultural role, and/or an economic role within ‘small-scale livelihoods’ involving agriculture, pastoralism, forestry, hunting and/or gathering (Trauernicht et al. 2015; Hoffman et al. 2021; Smith et al., 2022). By small-scale livelihoods we mean those relying predominantly on family labour or labour exchange with other households, rather than employment of labourers (Morton 2007). The purposes of controlled fires in this context are varied and numerous (e.g., clearing weeds or crop residues to facilitate new crops, enhancing forage for livestock) and often have multiple direct and indirect benefits (Laris, 2002). Such fires are our focus in this article, and we refer them hereafter as ‘small-scale livelihood and/or cultural fires’.”

*Our definition explicitly notes that we include labour exchange with other households in the concept of ‘small-scale livelihoods’, and that they ‘predominantly’ do not rely on employed labour, which therefore does not preclude cases where small-scale farmers hire additional workers at particular times of year. We also include traditional and Indigenous fire use, which is why we specifically use the term ‘small-scale livelihood and/or cultural fires’ – something that we also make clear in the abstract (“anthropogenic fires set to meet small-scale rural livelihood objectives and/or for cultural reasons”). We feel, therefore, that we provide reasonable clarity in defining the scope of the fire use that we are considering from the outset of the manuscript. For more in-depth discussion of the scope of the fire uses considered, the reader can further refer to the referenced Smith et al. (2022) paper that describes the LIFE database in more detail.*

*We are unsure how to engage with the reviewer’s comment querying how or why collecting more data will be useful. One of the objectives of the manuscript, as described in the abstract, is to highlight the limited data available on many types of anthropogenic fire use which precludes quantifying their contribution to observed fire regimes as well as representing them in models. This is discussed in more depth in the Discussion and Conclusion than is possible in the Abstract, inevitably, but nonetheless we would not think this to be an objectionable statement.*

*INTRODUCTION*

The connection between what the authors perceive as missing, and our ‘limited picture of possible future fire regimes and the adaptations we may need to make to ensure sustainable landscapes.” Is not made clear. E.g. How does “limited success in reproducing satellite-derived burned area estimates” connect to not being able to consider adaptations?

I find the ‘so what’ question of the introduction is left unstated and unclear.

*Thank you for this. We agree that we could have made our central argument more explicit. Briefly, our argument is that limited success in reproducing satellite-derived burned area estimates is symptomatic of flawed biophysically-oriented models – particularly regarding the human dimensions of fire regimes. The structural limitations in these models makes projecting how fire regimes will respond to future climate and socio-economic change challenging at best, which in turn makes understanding adaptation options in those largely unknown conditions more challenging still. Improved global scale models are not a panacea but can contribute to tackling this issue, alongside continued important local studies. We have revised the text to clarify the argument we are making at L80-90.*

METHODS

The data covering ~550 fires over 86 countries should be presented in table somewhere. What is the representation of the countries, are these cases equally spread? Beyond countries, we need to know also about the coverage of biomes and the role of fire in these biomes. For instance how are fire-sensitive or fire-adapted biomes represented?

*Please note that each record in the LIFE database – a ‘fire use practice’ – is not documenting a single fire, but is itself capturing the overall characteristics of numerous fires on the relevant study landscape. Figure 1 shows the geographic location of all the case studies on a map, while Table 2 presents a summary of the data which collates 587 different case studies, spanning 1648 different fire use practices (each of which represents numerous individual fires). The full data are available at the reference provided (Smith and Mistry (2022),* [*https://doi.org/10.17637/rh.c.5469993*](https://doi.org/10.17637/rh.c.5469993)*); this contains a spreadsheet which records the latitude and longitude, country, and biome for every case study location. As it consists of 1648 different entries, evidently, we could not reproduce the spreadsheet in the manuscript – and indeed, the purpose of the manuscript is to synthesise these data to try and draw some generalisable conclusions. Hence, as shown in Figure 1, we divide the world into three broad climate zones following Feddema (2005), based on temperature and precipitation seasonality, and show where each case study is located on this map, as well as summarising how many practices for each fire use purpose fall into the different climate zones (Table 2).*

*The role of anthropogenic fire in each case is implicit in the fire use purpose categorisation, which codifies the reason that fire is used, and it is evident from Table 2 that many fire use purposes were recorded preferentially in one or other of the climate zones. One thing that we commented on in the Discussion [L585-593 in revised text], as both a limitation and a conclusion, is indeed the uneven geographic distribution of the available case studies (as evident in Figure 1), meaning that certain regions of the world are better represented. A more systematic approach to anthropological studies of human fire use is precisely one of the outcomes we would like to see.*

*A more fine-grained categorisation of biome could be useful but would result in smaller sample sizes per biome / fire use purpose pairing. This would defeat the underlying focus of the manuscript – to assess the extent to which large-scale patterns do exist, which characterise some of the emergent properties of anthropogenic fire use. Furthermore, whilst fire-adapted and fire-prone biomes are well-established concepts, they are not compatible with dynamic global vegetation models. As such, to advance understanding in a way that contributes to fire science more broadly, we have adopted the simple climatic categorisation given here.*

*To that end, Smith et al. (2022) aggregated the 1648 different individual fire use practices by 8 higher-tier and 29 lower-tier fire use purposes, and we further aggregate by these three climate seasonality zones, and we show that for at least some fire use purposes there are, on average, some generalisable properties. Moreover, for the fire use purposes where no coherent patterns emerged, this was frequently due to a lack of data rather than because no such relationship exists. That many fire use purposes were very lacking in quantitative data, reinforces our decision to aggregate by a very minimal set of three climate zones, or in some cases purely by fire use purpose. Had we tried to aggregate by more fine-grained biomes, drawing any conclusions would have been impossible because all the sample sizes would be too small.*

*That we were able to draw out some broad explainable patterns in the timing, size, and frequency of fire use for those purposes where there was sufficient data, also validates this choice and shows that generalisable relationships do exist even when aggregating the data by a simple set of categories. For any readers who do want to see a list of all the biomes represented in the LIFE database in more detail than the map in Figure 1 can show, this is still available in the spreadsheet at the DOI provided for the dataset, which records the WWF biome category for every entry.*

Table 1. The higher use purpose category of Human wellbeing is not well articulated/bounded or defined. It includes many actions to reduce the risk of fire spread – to reduce flammability. If this constitutes well-being then just as easily many of the other categories could also be subsumed in HWB. For instance, fire for agriculture. Agriculture to feed people, thereby delivering to HWB. So, I think the current category of HWB needs unpacking and rethinking. Probably a Wildfire reduction, or Risk reduction category is required. Essentially HWB is the over-arching umbrella – the universal dimension to which all fire uses contribute for particular actors.

Similarly the discussion of co-benefits, and how they were defined or determined, and why they were left out of the spatio-temporal analysis is not explained. L146-153 and Table 1.

*Here, we are adopting the categories published in Smith et al., (2022; Nature Sustainability). No categorisation of human fire use is going to be perfect: a categorisation is in effect a mental model – and “all models are wrong”. In recognition of this, we present both the higher-tier fire use purposes (including “HW”) and the more granular lower-tier purposes (including e.g. HW2: fuel-load reduction, HW3: creating a firebreak, etc.), which are described in Table 1. The higher-tier purposes have larger data samples, but necessarily less ontological precision. The lower-tier purposes can be more precisely bounded but have smaller sample sizes. All of our subsequent analysis is in fact carried out on the more granular lower-tier purpose categories, not on the higher-tier purposes like “HW”. We have also added a new Appendix which describes each of the specific lower-tier categories in more detail, including the rationale for considering them as primary purposes or co-benefits.*

*We have added text to clarify why we subsequently chose not to present further analysis of those lower-tier fire use purposes which are typically co-benefits only [L308-314]:*

“The lower tier fire use purpose categories from Smith et al. include both primary, livelihood-oriented, reasons for fire use, such as driving game for hunting or clearing vegetation for swidden agriculture, as well as reasons that are usually co-benefits, such as reducing livestock pests, producing an aesthetically pleasing landscape, or maintaining cultural identity. Primary reasons are more likely to sustain fire use and influence the spatiotemporal patterning of fire (Bliege Bird & Bird, 2015; Current Anthropology), and so hereafter we do not present spatiotemporal data for those reasons we deem to usually be co-benefits only”.

L154. Fire purposes were derived by the source: ‘only when the purpose was stated in the source’. The authors must acknowledge the caveats around this. The source often being some form of ‘expert’ opinion or resource which quite likely reduces the complexity of fire use. For instance, one only has to look at the conservation literature to know that the purpose of fire is often given as ‘slash and burn’, when in reality fire use may have several other roles. We only have to think to the colonial policies that gave the purpose as laziness or recklessness etc to know that the top-down view of fire purpose can be erroneous. Hence, there is some caution that should be taken when extracting ‘purpose’ from these sources. Particularly those non-anthropological or social science sources.

Related to the above point, knowing the distribution of source types would also be useful. Specifically, the source type and its disciplinary lens.

*We share your concern, particularly where a fire serves multiple purposes and may not fit neatly into western ideas around rationality and decision-making. In the paper describing the LIFE database (i.e. Smith et al., 2022), exactly this concern is discussed. The distribution of source-types and methods in the LIFE database (e.g. ethnography, interviews, etc.) are also presented and discussed in the Smith et al. (2022) paper. However, disciplinary background of every case study is not something it is possible to assess objectively or systematically, and it is also no guarantee of precision and lack of bias. For those researchers that share the reviewer’s concerns around contributions from outside of the social sciences, it is possible to extract sources based on ethnography from the underlying data. Of course, this would then limit sample sizes further. This specific sentence has been removed in the re-written Methods section, and we now clarify and signpost more directly that we are drawing on the methods described in Smith et al. (2022) to assess fire use purposes [L131-133 and L151-156]:*

“When the database was created, separate fire practices were distinguished based on the criteria by which the author(s) of the source literature distinguished between distinct types of fire use – usually based on the purposes of fires (for more detail see Smith et al.)”

“Information about fire-use purposes was initially recorded in LIFE as descriptive free text information. Smith et al. used this information to create a hierarchical fire-use purpose classification including eight higher tier categories (agriculture, pastoralism, hunting and fishing, gathering, charcoal and firewood production, movement, promoting human health and wellbeing, and social signals) each of which is associated with several of 29 lower tier categories which we use again here (Table 1, with detailed descriptions given in S1 Appendix A)”.

L173-175: less than n 5 case studies for deriving the burned area info, and less than n5 for deriving the fire return info. This is indeed a very small sample. From which of the 86 countries and biomes do these derive? One must acknowledge that there is no representativeness here.

*We do indeed acknowledge this explicitly. Limited data is exactly why we don’t present quantitative results for those fire use purposes where there were 5 or fewer fire use practices in that category. We also note this issue in the title and abstract - “For many fire types, especially those related to hunting, gathering, human wellbeing, and social signalling, there are very limited quantitative data available”. Nonetheless, as described in L337-338 and L370-371, there are still several fire use purposes for which quantitative data was available from more than 5 fire use practices, and these are the ones we proceed to analyse.*

L177-179: what does ‘we selected one value’ mean here?

*Thanks for this clarifying question. Because quantitative fire information is not recorded consistently between sources, LIFE records the mean, median, minimum and maximum fire sizes where these are available. From these we selected the best available estimate of central tendency to allow for comparison between practices one value. We have revised the description in the Methods to try and clarify this [L197-201]:*

“…for each fire use practice for which quantitative data were available, we selected the best available estimate of central tendency to allow for comparison between practices. Where a mean or median value was available, we selected this, with the mean taking priority over the median where both were available. Failing that, we used the midpoint of the range, and failing that, the approximate value”.

*N.B. An approximate value arose, for example, if a source said, ‘burned areas are usually about 1 ha’, without qualifying how this number was arrived at.*

FIG 1. It is not possible to evaluate the figure because it is highly pixelated. However, the legend is misleading. So far as I understand it, burned area and return interval are taken from less than 5 case studies. This information should be given in the Figure legend. At present instead it cites the number of fire purposes associated with each which is higher (>100), which on one hand can make it appear the dataset is larger than it is (misleading), and on the other hand presents an issue because it suggests many purposes are recorded per site in a form of double counting. The categories in the legend do not match those explained above in the previous table.

*We’re sorry to hear about the pixelation of the figures – during submission we had to provide the figures as separate files, which were presumably then combined somehow in the version that was sent to reviewers; we had no control over this part of the process. The files were all uploaded in high resolution, but unfortunately it would appear the version you received was not the original full-size image.*

*Figure 1a shows the location of all the case studies for which BA data was available. Figure 1b shows the location of all the case studies for which return interval data was available. There are far more than 5 locations in both cases. It is correct that in many cases multiple fire use practices were described within the same case study, and these are therefore recorded as different entries in the database; this is described in the methods (“LIFE describes 1648 fire use practices which were recorded in 587 case study locations spanning 86 countries”). It is hopefully uncontentious that at the same case study location, people may set different types of fire, at different times of year, and to achieve different objectives. The purpose of our analysis is to aggregate fire uses by their intended purpose and not by their location – the latter is what the original case studies have inherently already done, by describing all the different fire uses at a single location. Here, we are interested in whether fires across different locations have similar properties depending on their intended purpose. Therefore, fires set for different objectives are considered as separate practices, and this is the reporting variable we are primarily interested in when assessing how well represented a given fire use purpose is, i.e. the ‘n’ reported in Table 2 and in the caption of Figure 1.*

*In any case though, it is incorrect that BA and return interval are taken from less than 5 case studies - which is why there are far more than 4 locations plotted in Figures 1a and 1b, and why multiple fire use purposes in Table 2 have n > 5 for BA and/or fire return interval.*

*We have revised the description in the Methods to now clarify how many distinct case study locations and countries are represented in the fire use practices for which we had quantitative information [L184-191 and L203-210]:*

“Quantitative measures of burned areas and fire return intervals associated with a fire use practice were recorded in LIFE as average values, minimum values, maximum values, and/or approximate values, depending on the kind of data available. At least one of these values was available for burned area for 165 fire use practices with known purpose(s) recorded at 113 case study locations spanning 40 countries, and for fire return interval for 298 fire use practices with known purpose(s) recorded at 210 case study locations spanning 49 countries. The geographical distribution of these case study locations are shown in Fig 1 a, b”

“Seasonality information was recorded in LIFE either in the form of months of burning (data available for 572 fire use practices with known purpose(s), spanning 233 case study locations across 57 countries), and/or qualitative descriptions of seasons of burning (data available for 715 fire use practices). …for our seasonality analysis here we only make use of the 572 fire use practices for which information was recorded in the form of months of burning. The geographical distribution of the 233 case study locations of these fire use practices for which months of burning were available is shown in Fig 1c”.

TABLE 2. Unclear why there are x2 n’s. The key gives a colour coded set of n’s which are presumably case study locations. Whilst the table gives another (undefined) set of n’s which are presumably individual burns. But it is not stated and is therefore not clear.

Most of the table is red, which indicates that the case studys are less than <6. The abstract of the paper over-sells. It does not suggest these small sample insights. Rather it foregrounds a large data set with over 500 entries. The authors should scale-back their pitch of the paper which is at odds with the delivery.

Further it is entirely unclear why the authors are seeking to aggregate this information across diverse contexts and biomes. Why attempt to determine a median fire return interval for less than 6 cases, the 6 of which are situated in different contexts in terms of the role and benefits (ecological as well as social) of fire? Some fire-adapted biomes would be expected to have higjer return intervals, whilst moist or tropical regions not.

*Here, we have to disagree with the reviewer. At no point do we analyse samples smaller than 6 cases: we explicitly remove these from our quantitative analysis. This is why they are coloured red in Table 2, and we don’t report quantitative values for these categories. We also explain this unambiguously in the caption for Table 2 (“Mean, median, and standard deviation (SD) are only provided where n≥6”). We have now further clarified this in the revised Results section text [L320-322]:* “We only report these statistics and further analyse those fire use purpose categories where there are at least 6 or more fire use practices for which we have a central estimate of burned area and/or fire return interval, or information on months of burning”*.*

*We don’t believe we oversell the analysis: “*gaps in data*” is a part of the paper’s title, after all. This is also why we say in the abstract,* “For many fire types, especially those related to hunting, gathering, human wellbeing, and social signalling, there are very limited quantitative data available”*. However, to avoid any potential confusion for this point, we have revised the abstract and methods [L33, L184-191, L203-210], to state the number of fire use practices and case study locations with relevant quantitative data that we use in this study, rather than the total number of case studies in LIFE as previously.*

*The “n’s” always refer to the number of fire use practices, as defined in the LIFE database. We have gone through and made some minor edits throughout the paper to ensure that we are consistent in our usage of this terminology. In particular, at no point do we associate “n” with individual burns. We have clarified in the caption of Table 2: “*‘n’ indicates the number of fire use practices with data contributing to the analysis. Mean, median, and standard deviation (SD) are only provided where n≥6*”. These descriptive statistics refer to the distribution of fires (not individual burns) reported for the different fire use practices. We have also revised the Methods to try and clarify the nature of the data being analysed [e.g. L131-133, L184-187]:*

*“*When the database was created, separate fire practices were distinguished based on the criteria by which the author(s) of the source literature distinguished between distinct types of fire use – usually based on the purposes of fires*”*

*“*Quantitative measures of burned areas (in hectares) and fire return intervals (in years) associated with a fire use practice were recorded in LIFE as average values (mean or median), minimum values, maximum values, and/or approximate values (where a single value was stated in the source without explanation of how it was derived), depending on the kind of data available*”.*

*Hence, for example, for swidden agriculture (purpose A1), there were n=99 fire use practices in the database with data on burned area, n=136 fire use practices with data on return interval, and n=144 fire use practices with data on seasonality. This is what Table 2 shows. Most of the aforementioned cells under the A1 purpose are coloured green because they have n>19, and this colour-coding is what the key describes. The exception is the temperature-driven seasonality zone, which had 0 case studies describing fire used in swidden agriculture, and so following the key this cell is therefore coloured red since n<6.*

*The reviewer is correct that most of Table 2 is red. This indicates that for the combination of that particular variable and that particular fire use purpose, there were fewer than 6 fire use practices recorded for which we had quantitative information. The fact that this is the sum of the quantitative information contained in an exhaustive search of 587 case studies is not an oversell: it’s the very point we are trying to make. I.e., we looked at all the case studies in the published literature, and yet for a lot of fire use purposes still could not find adequate information to draw quantitative conclusions.*

*As we have discussed in our earlier responses, the fact that there are less than 6 case studies for many of the possible combinations of fire use purpose and quantitative variable, does not detract from the fact that there are also a number of fire use purposes for which we *can* make quantitative statements about the typical burned areas, return intervals, and/or seasonal timing of fire use.*

*As noted above, in principle, of course, it would be interesting to look at more granular ecoregions. However, we don’t have the data to do this.*

L207-209. The extent to which this is double counted is unclear. A single location can have multiple purposes, yet numbers of ‘purposes’ is what is often cited. E.g. months of burning ((617 fire use practices) – but the real question is number of burns, and location of burns. In what systems are these burns occurring at particular times. I do not see how comparing everything with everything offers any value. Of course there are differences between them, and finding any pattern between these differences does not afford any insight. For instance, deriving seasonality for burn purposes when burns are scattered over biomes with different seasons and ecologies doesn’t bring much insight.

The authors later say they apply 3 climate zones as a way to cluster the data. However, the interplay of climate zone and ecology or biome is needed. For instance, there are parts of the Amazon that are savannah – but these would be included in the same climatic zone.

*The reviewer is raising an interesting question, but it is not one that can be answered with any existing data set. We do not know the number of burns. This could possibly be estimated by dividing the estimated burned area by fire size, but with the available data this would result in even smaller sample sizes still, given the few studies reporting both these metrics together. Further, we cannot detect fire counts from remote sensing (as discussed in the Introduction of the paper).*

*Therefore, the only present means of answering the reviewer’s question would be modelling, and dynamic global vegetation modelling in particular. Yet these global models do not currently explicitly represent intentional/managed human fire use and efforts to improve this representation (e.g. Perkins et al., (2024, GMD*, *https://doi.org/10.5194/gmd-17-3993-2024 ) do not currently attempt to capture seasonality.*

*Hence, before we can address the reviewer’s question, we need to find a way of systematically representing human fire use, and its seasonality, in global models, which is what we are working towards here. A combination of modelled burn counts, seasonality and case study data could indeed begin to address the reviewer’s question. However it will require a substantial endeavour to reach this point and not one that we can jump to in a single study.*

*Our analysis of seasonality uniformly adopts the 3 climate zones. It is not right to say that we “later” do this. We have described above our reasons for choosing this simple climatology: primarily a lack of data, but also a desire to integrate with global fire models (as discussed in the Introduction). Of course, with finer scale ecoregions, we could capture the kind of issue (Amazon savanna regions) the reviewer describes. With our approach, this would form part of an error term in the resulting seasonal distribution. Nevertheless, we are able to identify meaningful seasonal patterns from many fire use purposes. The reviewer is asking for additional precision that is not achievable with any existing dataset.*

*Let us restate our research aims to justify their importance. The question we are trying to answer with this manuscript is “whether different fire-use purposes can serve as indicators of the seasonal timing, fire return intervals, and burned areas of anthropogenic fires” [L114-116]. Consequently, the number of fire use practices across varied locations but with the same intended purpose is the metric we are interested in, and which is reported in Table 2. Whether different uses of fire occur at the same location is not a question we are trying to explore. It may separately be an interesting question, but it’s not our question here.*

*We are interested in whether small-scale livelihood and/or cultural fires set deliberately by humans across different locations have similar properties depending on their intended purpose. And we show that, yes, for at least some categories, the fire-use purposes can indeed serve as an indicator of the seasonal timing, fire return intervals and/or and burned area. From their own perspective and research priorities, the reviewer may not think this is an interesting or insightful result . However, there is a community of fire scientists – particularly those involved in large-scale modelling and/or Earth observation – who will also regard this as interesting and potentially useful for model development and interpretation and analysis of remote sensing results.*

L256-257: this is problematic given we know that fire use is intricately place based and dependent on many local climatic and ecological cues. Thus, burns do shift their timings depending on local realities, and not on anticipated norms or climatic averages as per the approach here.

*We would note that the climatological seasonal cycles are calculated at each individual location. But certainly, it is nonetheless problematic – local weather events and ecological cues of course influence the timing of burns from one year to the next as well. That the local climatological seasonal cycle captures enough information to be useful as an indicator of the timing of fire use is an assumption, and one that we are ultimately testing the validity of. As we show and explore in the Results, for some fire use purposes it works well, but for others it does not. We discuss some of the cases where this assumption doesn’t work well, and the reasons for it, in the Results, e.g. L472-476, L510-513.*

*It should also be noted that the time of burning is only recorded in LIFE at the calendar month level, and so any variation in timing of burning due to specific weather events is not captured in the underlying data in any case. Our analysis therefore only seeks to define the seasonality of burning at, at most, a monthly time resolution - we are not able to consider variation on shorter timescales than this. Moreover, across the various case studies in LIFE that recorded seasonality data, while some of them may have described the timing of burns during a particular year, other studies may have spanned multiple years or described practitioners’ typical patterns of burning – in which case, the climatological seasonal cycle is exactly the local climatic factor that would correlate with this. We therefore had to make a choice – using the weather data from one particular year is not obviously a better option, because the timings reported in a given case study might be for a ‘typical’ year. Hence, the assumption that we chose to make, as a hypothesis that the local climatological seasonal cycle is broadly related to the reported timing of burning – and which is borne out, at least for some fire use purposes.*

*We agree that local realities influence burning choices. These would form a part of the error term in our seasonal distributions. But this does not prevent us from seeking to generalise and therefore understand broader patterns. Of course, losing information from cross-case study comparison is inevitable and unavoidable. At present, there is no representation of seasonality of human fire use in global models at all. We propose an improvement, not a perfect solution, which would depend on large new datasets being compiled through systematic fieldwork. We have largely re-written the discussion/conclusions, and this point is now stated more prominently, e.g. L544-568 and L601-612.*

L262: an enormous amount of data shows that the reliability of seasonal shifts is changing inordinately in many regions.

*Yes, we address this point in the methods section at L261-264:*

*“*The choice of 1990 - 2020 to define this climatology aligns with the time range of the case studies included in the LIFE database. Although some shifts in regional weather patterns will inevitably have occurred over this time, any shorter time period would be inadequate to robustly characterise the climatological seasons*”.*

*In the revised Methods we have also added further clarification of the limited temporal resolution that we are analysing [L264-268]:*

*“*Additionally, the time of burning is only recorded in LIFE at the calendar month level, and so any variation in timing of burning due to specific weather events is not captured in the underlying data. Our analysis therefore only seeks to define the seasonality of burning at, at most, a monthly time resolution, and we expect that in general, typical seasonal cycles will not have robustly shifted over this period*”.*

*It is well established in the climate science community that at least a 30-year baseline is needed to robustly characterize the climate state; any variations observed on shorter timescales may just be a transient fluctuation and not a genuine shift in the regional climate. Of course, this still leaves open the criticism of the previous comment, that the time of burning in a given year is potentially more related to those transient fluctuations than to the typical climate – but we would refer back to our response to the previous comment in this regard.*

*Furthermore, we note once again that the seasonality in LIFE is recorded, at best, at a monthly level. The seasonal climatologies that we define for each case study location, are then in fact based on a 3-month running mean. For the above reasons, we do not anticipate that a shift in seasonal cycles by multiple months, on average, across numerous regions of the world is robustly detectable in a sub-30-year time period. However if there is such evidence, then we would certainly be keen to consider the implications of that for our analysis.*

L263: the reliance on number of fire use records should be amended. How many burns is this?

*As noted above, the reviewer is asking for data that is not available in the case studies that contribute to the LIFE database, and furthermore is not available in any current data source.*

L269: how were these validated?

*As described earlier in the methods, the precipitation data was taken directly from the ERA5 reanalysis, an extensively documented, well-validated, and ubiquitously used weather dataset that assimilates a wide range of observational data. Readers can consult the validation and quality assurance documented with the ERA5 product. Furthermore, as these are extensively documented and used data, there are some known limitations: we discuss these limitations and our measures to address them in the text [L243-252].*

L291: yet the authors already said their approach depends on theoretical averages rather than yearly realities. i.e. they are at the end of their own critique here.

*This is not accurate. We said we assume that the months of burning that are recorded in LIFE are often reporting typical times of year that burning takes place, and/or are informed by the typical seasonal cycle at that location. The typical seasonal cycle may well change in future. But, it requires longer than a 30-year time period to meaningfully identify such a shift. In L291 [L296-298 in revised text], we are talking about the possibility of the typical climate shifting in the future, consistent with this assumption, and not about the weather varying in one particular year.*

L290-293: Whilst obviously I agree that it would not be possible to compare timings based on calendar months, the reliance on seasonal cycles is also not robust because these variables are only part of the equation. As per previous comments, ecology/biome is equally important. Other factors also determine fire use – e.g. market demand, connectivity, degradation of area, policies or restrictions/rules around burning, availability of climate data etc etc.

*As per our response to previous comments, it seems like we are talking across purposes here. Certainly, we agree that other factors influence fire use – we specifically mention this in our concluding paragraph (“*Case studies demonstrate that the locations and timing of fire use are influenced, for instance, by vegetation type, topography, state and local governance, economic pressures, and traditional knowledge*”, L603-605 in revised text). At no point do we claim that the timing of fire use is purely a function of fire use purpose and local climate – that would be daft. But, as we have discussed already, we want to address whether there are nonetheless generalisable relationships between local climate and the seasonal timing of at least some types of fire use. And we show that there are. Such general patterns related to a minimal set of predictors have value, because although they do not explain the fine detail of individual uses of fire in individual years, they may still help to explain and to better represent in global models some broad aspects of human-driven fire regimes.*

*In the revised manuscript, we have made clear that we complement our quantitative findings with qualitative data in LIFE that may help explain within-sample variation for many of the reasons the reviewer describes [L303-306].*

RESULTS

*Thank you for these comments. We have comprehensively restructured the results section such that we avoid repetition and highlight what is more novel. As such, rather than describing fire use purposes in turn, we now group results by quantitative aspects of fire regimes (fire return interval, burned area, fire seasonality).*

The top-line/first-cited result is something already known the world over. That fire is used for agriculture does not seem the result to give centre stage here. What is it that this dataset has afforded that was not clear or well-known before?

*Thanks: see also our general comment above. We begin our Results of both fire return interval and burned area with comments on the limited available data. We also think this revision serves to highlight contrasting quantitative characteristics of differing fire use purposes more clearly.*

FIG 2 and 3. Fraction of records with fire use purpose I think is misleading. What is the number of locations? Fire use purpose is a form of double counting so far as I understand it. That is that more fire use purposes does not necessarily mean more representation in the data, because they may all be from the same place/burn or multiple burns in the same place.

*As we also discussed in our response to previous comments, these questions do not align with the nature of the dataset we use and the objective of our analysis. LIFE does not record individual burns – multiple burns at the same location for the same purpose would typically be recorded as a single ‘fire use practice’, together with the range of months in which those burns typically take place, the range of fire sizes, and the typical return interval, if those data were available. Furthermore, the number of fire use purposes at the same location is not relevant here as we are interested in the number of different practices where a given fire use purpose is relevant in that month of the year, and this is what the fractions indicate. I.e. for all examples of that fire use purpose, for how many fire use practices is burning done in that month of the seasonal cycle?*

L363. Perhaps this could be the first result to cite. It is interesting that agri fires are the smaller fires, and also likely means these are the less risky fires (i.e. less likely to escape and easier to control). Which says something about targeting fire management policy.

A central problem with the results is that they first state that each of the purposes that were already known indeed exist. Which is not really a novel contribution. The second issue is that they are written in a list-like manner, summarizing the information on each burn purpose one by one. Which get pretty dry. Further, it is still unclear how this information is useful, or indeed, the data on which it was derived (because of the issue above of reporting number of purposes which seems to exaggerate the data set).

*Thank you for this. As noted, we have substantially restructured the results section. Now, after a brief reflection on data limitations, we do indeed highlight such differences between fire use purposes as the major finding in each results subsection [L339-355, L372-387, L455-506].*

DISCUSSION

*Thank you for these comments. In response to these comments and those of reviewer #2, we have rewritten several sections of the discussion to be more precise about the implications of the results presented.*

That small scale agricultural fires are timed with the planting cycle does not merit a lead result (to come first in the discussion). This is known. What is the new insight here? That is what should be discussed first.

*Our headline result is that there are detectable differences in quantitative outcomes of different fire use purposes. The first sentence in the original discussion stated this as: “*Our analysis suggests that for small-scale livelihood and/or cultural fire use there are relationships between fire-use purpose and fire return interval, burned area and seasonality*”; in the revised discussion this has been re-phrased as: “*Our results identify relationships between the underlying purpose of small-scale livelihood and cultural fire use and the resulting fire return interval, burned area, and seasonal timing*”. We think this is an important finding and that the reviewer has at several points cast doubt on its possibility might tend to demonstrate this. The case of agricultural fires is then given as an example.*

L559-561: the authors need to explain how this ‘quantitiative’ understanding of fires “can help understand and interpret improved detection of fire”. The statement is made, but the links are not described. How can it?

*Thank you, we agree that this point should have been better elaborated. We have now revised this section to compare findings here with the GloCAB cropland burning remote sensing product [L529-543]. We contrast our results and point to areas where GloCAB may be improved in future versions.*

L562-565: this material was said already in the intro. Higher detection capacbility of small scale fires means that previous quantification of fires have been under-estimates. Yes, fine. But that was i) said in the intro, and ii) is not the focus of this present study. This study has not worked with finer resolution data or advanced the geospatial side of fire science. So, unclear why this also appears in the first para of the discussion. The key advance of the paper is not clear.

*Thanks - this content has been deleted and the Discussion revised such that it is more specific (e.g. L529-543 as discussed above).*

L574-575. HOW will it? Can they give an example? Are the fires here georeferenced as such that they can show that a fire detected by satellite and assumed to be due to X was in fact due to Y?

*Thanks - this content has similarly been deleted and the Discussion revised such that it is more specific. We make the argument [L529-543] that a global cropland burning algorithm such as GloCAB can*

1) *use case study data to compare results*
2) *use case study data to ensure different agricultural systems are captured*

L577: but how will it when this method here has not used uanced climate data and instead ascribed potential averages?

*We are unsure how to respond to this as we are not clear on what “nuanced climate data” and “potential averages” mean in this context. The reviewer seems to be focused on short-term fluctuations (year to year) and not on long term trend (over multiple decades), which as we have highlighted above, is more meaningful from a climate change perspective. As we described in the response to an earlier comment, the seasonal cycles we use are climatologically accurate for each case study location, based on historical reanalysis data. The climatological seasonal cycle at a given location may shift in the future, and a parameterisation of the seasonal timing of human fire use based on our method would be able to respond to that.*

L600. The emphasis of remote sensing products in the discussion seems peculiar given the paper was not a geospatial anlaysis. This comment on the underestimation of coarse models is now being said for the 3rd time and is redundant material.

*Thank you. This content has been revised substantially as above.*

L615. Millington is over cited, and if the work is so fundamental, it should be summarized here. Further, if the paper is about how this data can improve models, then that should be the starting point. What are the traits of exitsing models. What are their specifics, and their caveats. Once that is laid out, the authors could illustrate how their grounded data can add to the models. They could do so with text and with figures.

*We have removed some references to Millington, and note that it is discussed explicitly in the introduction [L95-99 in revised text]. We have also revised the Discussion substantially such that it focuses on a particular example of a shortcoming in fire models (i.e. getting seasonality wrong in sub-Saharan Africa), and argue our results point towards anthropogenic burning as a likely explanation of this [L544-558].*

L619: Exactly. Yet the abstract does not say this. The abstract stages it as a large dataset.

*This is not accurate – we did not use the word ‘large’ to describe the dataset we use anywhere in the manuscript. We state the number of observations available. In response to this and other comments we have now revised the abstract to refer instead to the number of records that contribute to our quantitative analysis, rather than the total number in the LIFE database: “*We examine data from 345 case study locations spanning 69 countries regarding return interval, area burned, and seasonality of anthropogenic fires set to meet small-scale rural livelihood objectives and/or for cultural reasons*”. We agree that this brings additional clarification to the extent of the dataset available, and the reader can make their own inference about whether this is large or otherwise. We have now rewritten the results such that each section highlights data limitations before presenting findings.*

*N.B. As described now in the Methods [Sect. 2.1], LIFE in total contains 1648 fire use practices that were recorded at 587 case study locations across 86 countries, taken from 593 literature sources. However, not all of these sources reported quantitative information, which is why our analysis can only make use of 345 of these case studies, as we now discuss more explicitly in the revised Methods [L135-140]. The frequent lack of quantitative information in case study data is one conclusion we sought to highlight.*

Ultimately, the key findings are not clear and neither their relevance. The discussion is centred on modelling, whilst the paper is descriptive about a data set. The dots are not connected. Further, the reason for aggregating the data from such diverse contexts is not clear. Finally, the data set appears far smaller than implied, and the distribution and representation is never made clear.

You could rework the paper and focus on the modelling argument more coherently. You could make the case for why current models are lacking or heavily caveated, then explain their data set and its attributes, and then define how and where these attributes might be integrated with models to improve their insights. At present it is a paper of two halfs. Intro and Discussion focused on models and geospatial data, and then the method and results about a list of fire purpose attributes. The connections are not made.

Another option would be to explore the fire specific contexts separately and in more depth. Reflecting on policy etc.

*Thank you. We have now revised the Introduction, Methods, Results, and Discussion substantially. We agree that the link between local case study data and larger-scale modelling needed to be made clearer. Furthermore, the restructuring of the Results has also greatly improved the flow and focus of the manuscript, and we are now more precise about the description of the dataset which hope has improved the clarity of the methodology.*

*We have strengthened the argument around the limitations of current models [L80-90], pointing out that existing fire-enabled global vegetation models do not account for human agency, rather just metrics of human presence and consumption. This then strengthens the rationale for considering fire use purposes globally.*

*Secondly, we have strengthened the model application part of the discussion, highlighting how a consistent model bias in seasonality could begin to be addressed by these results, as well as ongoing challenges in capturing fire size in global models [L544-558] . We also make the issue of fire purpose as a means of moving beyond GDP and population density more explicit [L559-568].*

*Following these suggested revisions to the structure of the paper, we hope the argument is now better connected: why should we aggregate across case studies? So that we can identify sources of model bias (as we now explicitly do in the discussion) and enable formulation of representations of different types of human fire use in global models that contribute to understanding climate and other environmental change.*

**Reviewer #2:**

**General comments**

This paper highlights a critical gap in current global estimates of burned area, in terms of exclusion of smaller fires that may be anthropogenic, and makes a strong argument for better understanding the social and ecological drivers of small-scale fires. The authors make the strong argument that there is a need to better quantify the influence of human fire activity on global fire regimes or estimates of burned area, and the spatio-temporal patterns of human fire use to inform interpretation of remote sensing data and broad-scale fire modelling. However, I’m not convinced that analyses presented in this paper address this gap, or if/how the results presented could be used to improve interpretation remotely sensed data or improve calibration of modelling tools, in practice. The results provide interesting descriptive statistics, e.g., mean/median burn area associated with a particular fire practice or purpose, such as swidden agriculture, but it’s not clear how the authors envision these results be applied in the context of analysing global burn estimates, or improving models, despite some strong, albeit general, statements to this effect. The analyses also don’t indicate if the values for each fire use (e.g., mean burn area) or suite of values are sufficiently/significantly different amongst uses to differentiate amongst these.

For example, in lines 559 – 561 they write “Understanding anthropogenic fire quantitatively like this will be important to help understand and interpret improved detection of small fires in global burned area products from remote sensing.” Is the authors’ argument that their results provide strong evidence that small fires linked to planting cycles should/could be identified as agricultural fires? And/or that remote sensing data collection should focus on identifying small fires during these known seasonal periods? What is the implication of this? How would these data be used to inform interpretation of burns of different sizes, seasonalities or fire return intervals? Similarly, they argue (lines 574-575) that “Datasets and analyses such as we have presented here will improve our ability to identify human-set fires and to understand their likely timing relative to climate and other drivers.” I’m not sure the analyses presented here provide a clear method or approach (or robust enough dataset, given the small sample sizes for most fire use purposes) for identifying human-set fires from global burned area databases/remotely sensed data (although lines 632-633 are one example of a specific recommendation).

Further, much of the discussion focusses on re-stating the arguments and evidence from the introduction, relating to the gaps in coarser resolution remote sensing products, rather than of this paper’s results and their specific implications. A key finding referenced towards the end is the lack of quantitative data on human fire use – I think this is actually really interesting and relevant and could be fleshed out more (what kind of data are missing, how could this be collected in future studies, reasons for qualitative vs quantitative imbalance, research needs etc).

In summary, I would like to see a stronger link drawn between the results presented here, and the implications in terms of establishing indicators for predicting or identifying human-set fires. If this is not the intention of the paper, then I would suggest reframing.

Some specific comments are included below.

*Thank you for these useful comments and suggestions. We agree that the link between our results and remote sensing / models (EO/ESMs) could be made more explicit. We also agree that the original discussion was repetitive of the introduction and hence have revised and refined most of the discussion so that we are making more specific links between our data and analysis and identified limitations in EO/ESMs.*

*These changes are made in the revised text at L529-543 for EO, and L544-568 for models. In particular, we highlight:*

- *Applications of our results to emerging global cropland fires products, including to sense check results, ensure training data are globally representative and to ensure ground truth data are chosen for relevant seasonal windows [L529-543].*
- *Applications of a seasonality scheme to address a consistent model bias (i.e. peaking burning in sub-Saharan Africa too late) [L544-555].*
- *Applications of our data to address issues in capturing global fire size distributions in models [L555-558].*
- *Two suggestions for simple next steps for model development based on these results [L559-568].*

*We also expand and reflect further on the challenges around systematically collecting quantitative case study data on human fire use [L587-597].*

**Specific comments**

Line 27 (abstract): should be “varying spatiotemporally”? (delete “in”?)

*Thanks for spotting this – yes, it should have been “varying spatiotemporally”, this has been corrected.*

Line 50: While there has been increasing interest in small-scale, and particularly Indigenous, fire use in the past 2 decades, there are key studies prior to the 1990s. See for e.g., Lewis, H.T. (1978b) Traditional Uses of Fire by Indians in Northern Alberta. Current Anthropology, 19(2): 401-402.

*This is a fair point; ultimately in the original compilation of the LIFE database a cut-off date had to be set for data gathering, and the focus was on capturing data on contemporary fire use practices.*

Line 88: For a recent review that quantified reported reasons/outcomes of Indigenous fire stewardship, see Hoffman et al. (2021). Conservation of Earth’s biodiversity is embedded in Indigenous fire stewardship. PNAS, 118(2)

*Thank you – we have added this reference.*

Line 117-118: Small-scale livelihoods has already been defined, this is repetition – suggest deletion

*We would tend to agree. However, a previous review, as well as reviewer #1 here have focused in on being precise on this definition. So, we would prefer to keep these lines [L124-128 in revised text] as a reminder when we pivot back to discussing the dataset, since it seems like it is helpful for some readers.*

Lines 119-120: Similarly, these #s (# of practices and case studies) have already been cited above. Suggest deleting from para above and keeping only in methods. Also, I am not quite clear on what a “fire use practice” refers to – I understand that this is different “fire use purpose”, but I’m not sure what constitutes a discrete practice as identified in the database (Different burn units/fires? Different methods of burning?). There are obviously multiple “fire use practices” included in numerous case studies, please clarify what these refer to. This is a key point to clarify given the analyses/quantitative measures are associated with these practices.

*Thank you – we agree that we were insufficiently clear in our definition and consistent in the usage of our own terminology, which has led to confusion for both reviewers. We have overhauled our description of the dataset in the Methods to try and better describe the specific content of the dataset, as well as signpost the more in-depth description in Smith et al. (2022). [L129-140, L184-190, L203-209 in revised text]:*

*“*The database draws on 593 literature sources written or published since 1995 that report on original empirical research carried out since 1990. When the database was created, separate fire practices were distinguished based on the criteria by which the author(s) of the source literature distinguished between distinct types of fire use – usually based on the purposes of fires (for more detail see Smith et al. (2022)). Fire use practices included in the database were practiced at the time of the original research (92 %) or within living memory of the research participants (8 %). For each practice, LIFE includes qualitative and/or quantitative information about up to 37 social and biophysical variables (depending on the types of data available in each source). In this study we analysed data about fire use purpose(s), months when burning takes place, fire return interval and burned area. The subset of fire use practices with known purpose(s) for which data was available for at least one of these spatiotemporal variables were described at 345 case study locations spanning 69 countries*”.*

*“*Quantitative measures of burned areas and fire return intervals associated with a fire use practice were recorded in LIFE as average values, minimum values, maximum values, and/or approximate values, depending on the kind of data available. At least one of these values was available for burned area for 165 fire use practices with known purpose(s) recorded at 113 case study locations spanning 40 countries, and for fire return interval for 298 fire use practices with known purpose(s) recorded at 210 case study locations spanning 49 countries”*.*

*“*Seasonality information was recorded in LIFE either in the form of months of burning (data available for 572 fire use practices with known purpose(s), spanning 233 case study locations across 57 countries), and/or qualitative descriptions of seasons of burning (data available for 715 fire use practices). To avoid possible subjectivity in the interpretation of qualitative descriptions of the season, for our seasonality analysis here we only make use of the 572 fire use practices for which information was recorded in the form of months of burning*”.*

Line 121: I realize the LIFE database itself is not the subject of this study, but I would like to know the rationale for only including studies from 1995/research from 1990. This may have been cited in the Smith and Mistry (2022) or Smith et al. (2022) papers that provide details on the database, but would be worth referencing here

*This was a decision taken (in the original compilation of the LIFE database) as the primary interest was to focus on contemporary fire use practices (i.e. those occurring under present day socio-economic conditions). It was necessary to set a limit at some year, because otherwise we would have been reviewing paleo literature etc. We accept this was essentially an arbitrary choice.*

*We have clarified (added the description ‘contemporary’) in our initial description of the database [L124-126]: “*The Livelihood Fire Database is a repository of information about contemporary landscape fire use practices worldwide that meet cultural objectives and/or objectives within ‘small-scale’ livelihoods*”.*

Line 132: The Hoffman et al paper cited above seems like a notable omission from this list. Are all the relevant studies reviewed in that study included in LIFE/captured in the updated literature search?

*Thank you for this. The original data gathering for LIFE occurred throughout 2020. The updated search we conducted was run through Web of Science, without looking for additional review papers to check. However, we would note that the (excellent) Hoffman et al. paper is focused on biodiversity, which is not a major focus of our work.*

*We have looked at the reference list from Hoffman et al. and identified 4 papers that would meet criteria for inclusion in LIFE that are not currently in the data set. We note these below. Of these, only two (Saha and Howe, and Darabant et al.) include quantitative information applicable for the analysis presented here. We will include these papers in a future LIFE update, thank you for your suggestion.*

- *Darabant, A. et al., (2016), Journal of Applied Ecology, 53(5), pp.1613-1622.*
- *García-Serrano, C.R. and Del Monte, J.P., 2004. Economic botany, 58(1), pp.58-71.*
- *Miltner, B.C. and Coomes, O.T., 2015. Agroforestry Systems, 89, pp.409-420*
- *Saha, S. and Howe, H.F., 2003. Ecology, 84(12), pp.3118-3123.*

Line 150-151: Could you clarify if you analyzed spatiotemporal data for reasons that are listed as “primary purpose or co-benefit”? (edit: I can see from Table 2 that you did, but worth referencing in text)

*Thanks – yes, we have updated the description in the main text accompanying Table 2 at the start of the Results section [L313-314]: “*hereafter we do not present spatiotemporal data for those reasons we deem to usually be co-benefits only (as indicated in Table 1)*”.*

Table 2: The use of mean and median at various points in this table and the description of your analysis methods is a bit hard to follow, I had to re-read quite a few times to understand. If I understand it right, to get to the mean and median under “mean, median, midpoint of range, or approximate value”, you identified one of these values from each of the relevant studies (either a mean value, a median value, a midpoint of range, or approximate) and then calculated the mean and the median of these collective values? Perhaps just some minor tweaking to make this more explicit, e.g., calculating mean/median of these combined values

*We appreciate the reviewer for taking the time to understand what we did – yes this is correct, but fully accept our original description was a bit hard to follow! We have revised the description of the burned area and fire return interval data in the Methods section as follows, to try and make it more explicit by introducing the terminology of ‘the best available estimate of central tendency’ [L184-201]:*

*“*Quantitative measures of burned areas and fire return intervals associated with a fire use practice were recorded in LIFE as average values (mean or median), minimum values, maximum values, and/or approximate values... Where minimum and maximum values were available, we calculated the range. Very few sources provided average values for burned area or fire return interval. For all but three of our fire-use purpose categories, there were five or fewer fire use practices with mean or median burned area data. For all but one of our fire-use purpose categories, there were five or fewer fire use practices with mean or median fire return interval data. Given these limitations, for each fire use practice for which quantitative data were available, we selected the best available estimate of central tendency to allow for comparison between practices. Where a mean or median value was available, we selected this, with the mean taking priority over the median where both were available. Failing that, we used the midpoint of the range, and failing that, the approximate value*”.*

*And then, in the Results section where we now describe Table 2 [L316-322]:*

*“*For each fire-use purpose category, we calculated a mean, median, and standard deviation of the estimates of central tendency for burned area and fire return interval (Table 2). For each fire-use purpose category, we also calculated the mean, median, and standard deviation of the ranges of values for individual fire use practices (Table 2). We only report these statistics and further analyse those fire use purpose categories where there are at least 6 or more fire use practices for which we have a central estimate of burned area and/or fire return interval, or information on months of burning*”.*

Table 2: I would separate the seasonality section into a separate table. These are reporting very different values, and depending where the table is formatted in the final manuscript it may cause some confusion. Also, the caption for Table 2 does not reference seasonality

*Thank you for spotting the omission in the table caption – this has now been updated. We prefer to keep Table 2 structured as is since it serves to summarise the data availability of the three quantitative variables (burned area, return interval, months of burning) in one place, before we consider each one of these in turn in the Results, which is now completely restructured to have a separate section on each variable, rather than separate sections for each fire use purpose. We hope that the Table (now at the start of the Results section) therefore fits a bit more logically. (Splitting it would also result in a somewhat elongated 32 x 4 table for seasonality, which may be hard to format nicely).*

Section 2.3 and Table 2: Keep Section 2.3 focussed on your methods, avoid reporting results. Similarly, Table 2 seems like it would fit better in results.

*Thank you for this suggestion – as suggested we have moved Table 2 and associated text into the Results section, as part of a substantial re-structuring of the Results as well as re-writing parts of the Methods section. We fully agree this is a much better structure for the paper, and it is now more focused and easier to follow.*

Table 2: Please change the colours to be colour blind friendly. The darker green and orange appear the same to people with red-blind/protanopia and green-blind/deuteranopia conditions. There are numerous tools online you can use to check, or colourblind friendly palettes (e.g., in various software packages)

*Many thanks for highlighting this omission – we have updated and tested the colour scheme of Table 2 to now be colour blind friendly.*
